# Supplementary material for: Resolving the full spectrum of human genome variation using Linked-Reads
Source: Genome Res. 2019 Apr;29(4):635–45. doi: 10.1101/gr.234443.118 (PMC6442396; doi:10.1101/gr.234443.118)
Supplement: Supplemental Material [file supp_gr.234443.118_Supplemental_longranger-2.2.zip › longranger-2.2.tar.bz2!/lr2.2-public/lib/stan/cmdstan/stan/doc/api/html/structstan_1_1gm_1_1var__decl.html]

Stan: stan::gm::var\_decl Struct Reference


|  |  |
| --- | --- |
|  | Stan  2.5.0  probability, sampling & optimization |


- Main Page
- Related Pages
- Namespaces
- Classes
- Files

- Class List
- Class Index
- Class Hierarchy
- Class Members

All Classes Namespaces Files Functions Variables Typedefs Enumerations Enumerator Friends Macros Pages

- stan
- gm
- var\_decl

Public Types |
Public Member Functions |
Public Attributes |
List of all members

stan::gm::var\_decl Struct Reference

`#include <ast.hpp>`

|  |  |
| --- | --- |
| Public Types | |
| typedef boost::variant  < boost::recursive\_wrapper  < nil >  , boost::recursive\_wrapper  < int\_var\_decl >  , boost::recursive\_wrapper  < double\_var\_decl >  , boost::recursive\_wrapper  < vector\_var\_decl >  , boost::recursive\_wrapper  < row\_vector\_var\_decl >  , boost::recursive\_wrapper  < matrix\_var\_decl >  , boost::recursive\_wrapper  < simplex\_var\_decl >  , boost::recursive\_wrapper  < unit\_vector\_var\_decl >  , boost::recursive\_wrapper  < ordered\_var\_decl >  , boost::recursive\_wrapper  < positive\_ordered\_var\_decl >  , boost::recursive\_wrapper  < cholesky\_factor\_var\_decl >  , boost::recursive\_wrapper  < cholesky\_corr\_var\_decl >  , boost::recursive\_wrapper  < cov\_matrix\_var\_decl >  , boost::recursive\_wrapper  < corr\_matrix\_var\_decl > > | var\_decl\_t |
|  | |

|  |  |
| --- | --- |
| Public Member Functions | |
|  | var\_decl () |
|  | |
|  | var\_decl (const var\_decl\_t &decl) |
|  | |
|  | var\_decl (const nil &decl) |
|  | |
|  | var\_decl (const int\_var\_decl &decl) |
|  | |
|  | var\_decl (const double\_var\_decl &decl) |
|  | |
|  | var\_decl (const vector\_var\_decl &decl) |
|  | |
|  | var\_decl (const row\_vector\_var\_decl &decl) |
|  | |
|  | var\_decl (const matrix\_var\_decl &decl) |
|  | |
|  | var\_decl (const simplex\_var\_decl &decl) |
|  | |
|  | var\_decl (const unit\_vector\_var\_decl &decl) |
|  | |
|  | var\_decl (const ordered\_var\_decl &decl) |
|  | |
|  | var\_decl (const positive\_ordered\_var\_decl &decl) |
|  | |
|  | var\_decl (const cholesky\_factor\_var\_decl &decl) |
|  | |
|  | var\_decl (const cholesky\_corr\_var\_decl &decl) |
|  | |
|  | var\_decl (const cov\_matrix\_var\_decl &decl) |
|  | |
|  | var\_decl (const corr\_matrix\_var\_decl &decl) |
|  | |
| std::string | name () const |
|  | |

|  |  |
| --- | --- |
| Public Attributes | |
| var\_decl\_t | decl\_ |
|  | |

## Detailed Description

Definition at line 592 of file ast.hpp.

## Member Typedef Documentation

typedef boost::variant<boost::recursive\_wrapper<nil>, boost::recursive\_wrapper<int\_var\_decl>, boost::recursive\_wrapper<double\_var\_decl>, boost::recursive\_wrapper<vector\_var\_decl>, boost::recursive\_wrapper<row\_vector\_var\_decl>, boost::recursive\_wrapper<matrix\_var\_decl>, boost::recursive\_wrapper<simplex\_var\_decl>, boost::recursive\_wrapper<unit\_vector\_var\_decl>, boost::recursive\_wrapper<ordered\_var\_decl>, boost::recursive\_wrapper<positive\_ordered\_var\_decl>, boost::recursive\_wrapper<cholesky\_factor\_var\_decl>, boost::recursive\_wrapper<cholesky\_corr\_var\_decl>, boost::recursive\_wrapper<cov\_matrix\_var\_decl>, boost::recursive\_wrapper<corr\_matrix\_var\_decl> > stan::gm::var\_decl::var\_decl\_t

Definition at line 607 of file ast.hpp.

## Constructor & Destructor Documentation

|  |  |  |  |  |
| --- | --- | --- | --- | --- |
| stan::gm::var\_decl::var\_decl | ( |  | ) |  |

Definition at line 1208 of file ast\_def.cpp.

|  |  |  |  |  |  |
| --- | --- | --- | --- | --- | --- |
| stan::gm::var\_decl::var\_decl | ( | const var\_decl\_t & | *decl* | ) |  |

Definition at line 1207 of file ast\_def.cpp.

|  |  |  |  |  |  |
| --- | --- | --- | --- | --- | --- |
| stan::gm::var\_decl::var\_decl | ( | const nil & | *decl* | ) |  |

Definition at line 1209 of file ast\_def.cpp.

|  |  |  |  |  |  |
| --- | --- | --- | --- | --- | --- |
| stan::gm::var\_decl::var\_decl | ( | const int\_var\_decl & | *decl* | ) |  |

Definition at line 1210 of file ast\_def.cpp.

|  |  |  |  |  |  |
| --- | --- | --- | --- | --- | --- |
| stan::gm::var\_decl::var\_decl | ( | const double\_var\_decl & | *decl* | ) |  |

Definition at line 1211 of file ast\_def.cpp.

|  |  |  |  |  |  |
| --- | --- | --- | --- | --- | --- |
| stan::gm::var\_decl::var\_decl | ( | const vector\_var\_decl & | *decl* | ) |  |

Definition at line 1212 of file ast\_def.cpp.

|  |  |  |  |  |  |
| --- | --- | --- | --- | --- | --- |
| stan::gm::var\_decl::var\_decl | ( | const row\_vector\_var\_decl & | *decl* | ) |  |

Definition at line 1213 of file ast\_def.cpp.

|  |  |  |  |  |  |
| --- | --- | --- | --- | --- | --- |
| stan::gm::var\_decl::var\_decl | ( | const matrix\_var\_decl & | *decl* | ) |  |

Definition at line 1214 of file ast\_def.cpp.

|  |  |  |  |  |  |
| --- | --- | --- | --- | --- | --- |
| stan::gm::var\_decl::var\_decl | ( | const simplex\_var\_decl & | *decl* | ) |  |

Definition at line 1216 of file ast\_def.cpp.

|  |  |  |  |  |  |
| --- | --- | --- | --- | --- | --- |
| stan::gm::var\_decl::var\_decl | ( | const unit\_vector\_var\_decl & | *decl* | ) |  |

Definition at line 1215 of file ast\_def.cpp.

|  |  |  |  |  |  |
| --- | --- | --- | --- | --- | --- |
| stan::gm::var\_decl::var\_decl | ( | const ordered\_var\_decl & | *decl* | ) |  |

Definition at line 1217 of file ast\_def.cpp.

|  |  |  |  |  |  |
| --- | --- | --- | --- | --- | --- |
| stan::gm::var\_decl::var\_decl | ( | const positive\_ordered\_var\_decl & | *decl* | ) |  |

Definition at line 1218 of file ast\_def.cpp.

|  |  |  |  |  |  |
| --- | --- | --- | --- | --- | --- |
| stan::gm::var\_decl::var\_decl | ( | const cholesky\_factor\_var\_decl & | *decl* | ) |  |

Definition at line 1219 of file ast\_def.cpp.

|  |  |  |  |  |  |
| --- | --- | --- | --- | --- | --- |
| stan::gm::var\_decl::var\_decl | ( | const cholesky\_corr\_var\_decl & | *decl* | ) |  |

Definition at line 1220 of file ast\_def.cpp.

|  |  |  |  |  |  |
| --- | --- | --- | --- | --- | --- |
| stan::gm::var\_decl::var\_decl | ( | const cov\_matrix\_var\_decl & | *decl* | ) |  |

Definition at line 1221 of file ast\_def.cpp.

|  |  |  |  |  |  |
| --- | --- | --- | --- | --- | --- |
| stan::gm::var\_decl::var\_decl | ( | const corr\_matrix\_var\_decl & | *decl* | ) |  |

Definition at line 1222 of file ast\_def.cpp.

## Member Function Documentation

|  |  |  |  |  |
| --- | --- | --- | --- | --- |
| std::string stan::gm::var\_decl::name | ( |  | ) | const |

Definition at line 1224 of file ast\_def.cpp.

## Member Data Documentation

var\_decl\_t stan::gm::var\_decl::decl\_

Definition at line 609 of file ast.hpp.

---

The documentation for this struct was generated from the following files:

- src/stan/gm/ast.hpp
- src/stan/gm/ast\_def.cpp


---

[ Stan Home Page ]

*© 2011–2014,
Stan Development Team.*
